# Poor and non-poor gap in under-five child nutrition: a case from Nepal using Blinder-Oaxaca decomposition approach

**DOI:** 10.1186/s12913-022-08643-6

**Published:** 2022-10-12

**Authors:** Umesh Prasad Bhusal

**Affiliations:** 1 Public Health and Social Protection Professional, Kathmandu, Nepal; 2 Melbourne School of Population and Global Health, The University of Melbourne, Melbourne, VIC Australia

**Keywords:** Child nutrition, Gap, Inequality, Blinder-Oaxaca decomposition, Height-for-age z scores (HAZ), MICS, Nepal, SDGs, UHC

## Abstract

**Introduction:**

Many low-and middle-income countries (LMICs) have improved health indicators in the past decades, however, there is a differential in outcomes between socioeconomic groups. Systematic analysis of drivers of child nutrition gap between non-poor and poor groups has a policy relevance in Nepal and other countries to make progress towards universal health coverage (UHC). The objective of this paper was to estimate the mean height-for-age z scores (HAZ) gap between under-five children belonging to non-poor and poor groups, divide the gap into components (endowments, coefficients and interaction), and identify the factors that contributed most to each of the component.

**Methods:**

Information about 6277 under-five children was extracted from the most recent nationally representative Nepal Multiple Indicator Cluster Survey (MICS) 2019. HAZ was used to assess nutritional status of children. Wealth index was used to categorize children into non-poor and poor. Mean HAZ gap between groups was decomposed using Blinder-Oaxaca technique into components: endowments (group difference in levels of predictors), coefficients (group difference in effects of predictors), and interaction (group difference due to interaction between levels and effects of predictors). Detailed decomposition was carried out to identify the factors that contributed most to each component.

**Results:**

There was a significant non-poor and poor gap in nutrition outcome measured in HAZ (0.447; *p* < 0.001) among under-five children in Nepal. The between-group mean differences in the predictors of study participants (endowments) contributed 0.210 (47%) to the gap. Similarly, the between-group differences in effects of the predictors (coefficients) contributed 0.308 (68.8%) towards the gap. The interaction contributed -0.071 (15.8%) towards minimizing the gap. The predictors/variables that contributed most towards the gap due to (i) endowments were: maternal education, province (Karnali, Sudurpaschim, Madhesh), residence (rural/urban), type of toilet facility and ethnic group (Dalit and Muslim); (ii) coefficients were: number of under-five children in family, ethnic group (Dalit and Muslim), type of toilet facility, maternal age and education.

**Conclusion:**

Decomposition of the child nutrition gap revealed that narrowing the inequality between wealth groups depends not only on improving the level of the predictors (endowments) in the poor group but also on reducing differential effects of the predictors (coefficients).

**Supplementary Information:**

The online version contains supplementary material available at 10.1186/s12913-022-08643-6.

## Background

Improving equality is one of the outcomes of health systems depicted in World Health Organization (WHO) health system framework [1]. It is one of the goals inherent in the concept of universal health coverage (UHC) [2]. Many countries in low-and middle-income countries (LMICs) have improved their health indicators in the past decade, however, there is a differential in outcomes between groups based on gender, ethnicity, geography, and socioeconomic status [3–9]. To meet the commitment of achieving UHC (where people have access to needed quality health services without financial hardship) by 2030 [10], countries are continuously reframing their policies and working towards narrowing down the health gap between various groups. This has called for global and country-specific studies focused on the status and drivers of the health gap.

A growing body of literature from LMICs has reported a significant gap in under-five child nutrition status between wealth groups [3, 5, 11–16]. Children belonging to poor families are reported to have worse nutritional outcomes compared to their non-poor counterparts [11–14, 17]. Systematic differences in the distribution of predictors (such as age of child, sex of child, education of mother, age of mother, household sanitation, ethnic group, geographical location) across non-poor and poor groups contributed significantly to explaining the gap in the nutritional outcome [11, 14, 17]. Similarly, the differential effects of such predictors between groups also contributed substantially towards the gap [11, 14, 17].

Child undernutrition is a major public health problem globally, including in Nepal. In 2020, an estimated 149 million under-five children worldwide were affected by stunting (too short for age), about half of them belonging to Asian countries [18]. Child undernutrition is linked to about half of deaths among under-five children globally [19]. Nepal has witnessed a remarkable improvement in child nutritional status in the last two decades. The proportion of children stunted (too short for age) has decreased from 57% in 1996 [20] to 31.5% in 2019 [21]. However, the progress is not up to the mark with the targets set by World Health Assembly that aimed to reduce stunting by 3.9% per year or 40% by 2025 from the baseline of 2010 [22]. Further, the evidence from the analysis based on Demographic and Household Surveys showed that the progress in child nutrition outcomes in Nepal was not equitable across wealth groups, instead, the wealth-related inequalities deteriorated between 1996 to 2016 [23]. Other studies have also demonstrated a significant disparity in the improvement of child nutrition indicators in lower economic groups compared to higher economic groups in Nepal [20, 21, 24–26]. Possible reasons for these continued disparities could be the government's attention largely focused on attaining the public health targets at the national level, without providing due consideration to monitor disaggregated figures across socioeconomic groups [8].

So, the systematic analysis of drivers of child nutrition gap between socioeconomic groups has a policy relevance in Nepal and similar countries to make progress towards UHC. Recent federalization of the country into three tiers of government (federal, provincial and local) in 2017 from the previous unitary system and equality as one of the principles reflected in national health policy 2019 has opened avenues for evidence-informed interventions targeted to narrow down the health gap across socioeconomic groups. In addition, the government of Nepal has pledged to improve the nutritional condition of children and achieve Sustainable Development Goal (SDGs) targets by 2030 [27]. More specifically, target 2.2 of SDGs is to end all forms of malnutrition by 2030 [28]. Nutrition-specific policies and strategies of Nepal are shaped not only by the National Health Policy 2019 but also by National Nutrition Strategy 2020, Multi-Sector Nutrition Plan II (2018–2022), and Nepal Health Sector Strategy (2016–2022). In all of the above policy documents, emphasis has been given to multi-sectoral actions to address the broader determinants of child nutrition to achieve equitable progress in nutrition outcomes. Specifically, National Nutrition Strategy aims to tackle all forms of malnutrition by providing health sector leadership towards nutrition-specific and sensitive interventions [27]. Whereas, Multi-Sector Nutrition Plan provides a broader national policy framework for nutrition interventions both within and beyond the health sector [27].

To formulate appropriate interventions against the health gap between wealth groups and monitor progress in narrowing the inequality over time, it is imperative to identify the sources of the health gap and quantify their contributions to the gap. Investigating to what extent the non-poor and poor gap in child nutrition is due to differences in the magnitudes of the covariates (predictors of child undernutrition) or due to differences in the effects of these covariates is crucial for formulating appropriate policy measures aiming at narrowing inequality and to make progress against the set national and international targets [29]. However, very few studies have analyzed the gap in child nutrition outcomes based on wealth status and investigated the sources of inequalities and their exact contributions [23, 30].

To address this research gap, and to contribute to the literature on health inequality, this paper sets the following objectives: (i) to calculate the mean height-for-age z scores (HAZ) gap between non-poor and poor groups; (ii) to identify the relative contribution of difference in the distribution of the predictors between groups towards to the gap; (iii) to identify the relative contribution of difference in the effects of the predictors between groups towards to the gap; (iv) to quantify the contribution of each factor to the gap obtained in (ii) and (iii) so that the factors that contribute most towards creating health divide can be identified. Blinder-Oaxaca decomposition technique was used to meet the above objectives [31]. To the best of our knowledge, there exists no such study from Nepal that has systematically decomposed the child nutrition gap into components with a clear contribution from different factors. The rationale of this study is that the evidence obtained from the above specific objectives will be helpful to policymakers and planners in Nepal to design the interventions targeted to poor families so that the SDG of narrowing inequality by 2030 could be achieved, together with the target of ending all forms of malnutrition and achieving UHC.

## Methods

### Data source and sampling design

This study was based on the most recent round of the nationally representative cross-sectional household survey, Nepal Multiple Indicator Cluster Survey (MICS) 2019. The survey, conducted jointly by Central Bureau of Statistics and UNICEF, aimed to track the situation of women and children by collecting data on health, education, social protection, environment along with socioeconomic and demographic characteristics of individuals and households. The sampling frame of the 2019 survey round was based on a list of all census wards constructed for the National Population and Housing Census 2011 (updated in 2018 after federalization in Nepal). Nepal MICS 2019 used a multistage, stratified, cluster probability sampling design to establish a representative sample of households at the national and province levels. Within each province, the main sampling strata were defined as urban and rural areas. The sample of households was obtained as follows: (i) a specified number of clusters or census enumeration areas (EAs) were selected systematically with probability proportional to size in each stratum, followed by household listing in selected EAs (ii) households’ sample was drawn from the sampled EAs (25 households per EA) using systematic random sampling. In this round of the survey, 12,800 households were selected from a total of 512 EAs. From this; 12,655 households, 14,805 women (15–49 years) and 5501 men (15–49 years) were successfully interviewed. Further detail about the survey design is available in the Nepal MICS report [21].

### Study population

For this study, data about HAZ of 6469 children under-five years were extracted from the Nepal MICS 2019 dataset. The data were obtained by interviewing mothers or primary caretakers of the children. The final analysis was based on 6288 under-five children (weighted count = 6277) after removing 181 observations with incomplete information. We assumed that the information was missing at random [32].

### Dependent variable

HAZ of under-five children (0–59 months) measured on a continuous scale was selected as the dependent variable. Height-for-age provides a measure of children’s height relative to their age (cumulative linear growth). It is responsive to long-term nutritional deprivation and chronic or frequent illness [33]. In contrast, the body weight of children is sensitive to short-term variations in nutrition and illness [34]. Therefore, HAZ is more commonly used as an indicator of child nutrition or chronic undernutrition [35]. It is calculated by dividing the difference between a child’s height and the median value for the reference population for the corresponding age and sex by the standard deviation (SD) of the reference population [33]. Nepal MICS 2019 used WHO growth standards as the reference population.

### Independent variables

An extensive review of the literature, including the framework on child nutrition, was performed to identify potential policy-relevant variables that were commonly used in anthropometric regression to study the determinants of children’s nutritional status [4, 11, 14–16, 36]. Nepal MICS 2019 dataset was explored to list the possible candidate variables that could be considered for our study (Supplementary Table 1). The list of candidate variables for under-five child nutrition was, thus, constrained by their availability in the MICS dataset. The multicollinearity among candidate variables was investigated to remove variables (preceding birth interval) that had a very high value of variance inflation factor (VIF > 10) [37]. Further, the forward selection method of variable selection using Akaike’s information criteria (AIC) as described by Lindsey and Sheather [38] was applied to identify the regression model that best fits the data. The variables included in the decomposition model were: child’s age (age of children in months); child’s sex (female, male); mother’s age at birth (less than 20 years, 20–34 years, more than 34 years); maternal education (no formal education, primary education (grade1-5), secondary education (grade 6–10), higher secondary and above (grade 11 and above)); number of under-five children in household (one, two, three, four or more); type of toilet facility (improved, unimproved); ethnic group (Brahmin/Chhetri/Madhesi, Janajati/Newar, Dalit/Muslim, Others eg. Marwadi/Bangali); residence (rural, urban); province (Province 1, Madhesh, Bagmati, Gandaki, Lumbini, Karnali, Sudurpaschim); wealth group (non-poor, poor). WHO guideline was used to categorize the toilet facility into improved and unimproved [39]. Population Monograph of Nepal 2014 was used as a reference to recategorize more than 100 castes listed during the survey into four categories [40]. VIFs of the variables included in the final model is provided as Supplementary Table 2. The non-poor and poor groups were created by recategorizing the wealth index quintile available in the dataset. The wealth index provides an economic rank to the households and is widely used in the literature as a proxy measure of living standards in absence of household data on consumption, income or wealth [11]. In consistence with the earlier studies [5, 11, 17], we re-grouped the bottom two quintiles (poorest and poorer) as poor and the upper three quintiles (middle, richer and richest) as non-poor. MICS employed principal components analysis to construct the wealth index quintile using the information on the ownership of goods, housing characteristics, water, sanitation, and other assets/durables that represent the household’s wealth [21].

### Method of analysis

Descriptive analyses were performed to compare the mean HAZ between the non-poor and poor groups across the independent variables. All analyses were conducted as per complex survey design to adjust for sampling weights, clustering and stratification in the sampling design.

### Blinder-Oaxaca decomposition method

The Blinder-Oaxaca decomposition method [41–43] was used to determine the factors that contributed to the mean difference in HAZ between the non-poor and poor groups. The following regression model was constructed, as given in Eq. 1

1 $$\Delta \overline{Y}={(\beta }_{0}^{np}-{\beta }_{0}^{p})+\sum_{i=1}^{k}({\beta }_{i}^{np}{\overline{x}}_{i}^{np}-{\beta }_{i}^{p}{\overline{x}}_{i}^{p})$$

where, $\overline{x}$ denotes mean value of each predictor variable (covariate); $\beta$ denotes estimated regression coefficient;‘np’ denotes ‘non-poor group’; ‘p’ denotes ‘poor group’; $(\Delta \overline{Y)}$ denotes predicted mean difference in HAZ between non-poor and poor groups.

The Blinder-Oaxaca decomposition method adopts a counterfactual approach that involves replacing the coefficients and variable levels of one group with the corresponding values of another group (reference group). In our analyses, we specified the non-poor group as a reference group to get the expected change in predicted mean outcome when the poor group gets the predictor values and regression coefficients from the non-poor group. The decomposition model as given in Eq. 2 was specified,

2 $$\Delta \overline{Y}={(\beta }_{0}^{np}-{\beta }_{0}^{p})+\sum_{i=1}^{k}{\beta }_{i}^{p}\left({\overline{x}}_{i}^{np}-{\overline{x}}_{i}^{p}\right)+\sum_{i=1}^{k}{\overline{x}}_{i}^{p}\left({\beta }_{i}^{np}-{\beta }_{i}^{p}\right)+\sum_{i=1}^{k}\left({\overline{x}}_{i}^{np}-{\overline{x}}_{i}^{p}\right)({\beta }_{i}^{np}-{\beta }_{i}^{p})$$

The decomposition model in Eq. 2 was constructed from the perspective of the poor group, where the non-poor group was specified as the reference. Here, the predicted mean difference $(\Delta \overline{Y)}$ of HAZ consisted of four components, as given on the right-hand side of the equation.

(i) First component provided the effects of unobserved characteristics that were not taken into account.
(ii) Second component provided changes in the poor group’s mean predicated value when it got the non-poor group’s covariates level. It yielded the portion of the predicted mean difference $(\Delta \overline{Y)}$ that could be explained by the group difference in the level of independent variables included in the model. This portion is referred to as ‘explained component’ or ‘endowments effect’ in the literature.
(iii) Third component denoted the changes in the poor group’s mean predicated value when it got the non-poor group’s regression coefficients. It involved the fraction of the predicted mean difference $(\Delta \overline{Y)}$ that was due to the differential effect of the covariates on outcome across non-poor and poor groups. This portion is referred to as ‘unexplained component’ or ‘coefficients effect’ in the literature.
(iv) Fourth component entailed an interaction caused by the simultaneous effect of differences in explained (endowments) and unexplained (coefficients) components.

Since the first component (i) dealt with differences between two groups that could not be explained by the covariates included in the model and the third component (iii) also dealt with an unexplained part of the difference, both components were combined to construct the three-fold decomposition model [31, 41], as given in Eq. 3

3 $$\Delta \overline{Y}=\sum_{i=1}^{k}{\beta }_{i}^{p}\left({\overline{x}}_{i}^{np}-{\overline{x}}_{i}^{p}\right)+\sum_{i=1}^{k}{\overline{x}}_{i}^{p}\left({\beta }_{i}^{np}-{\beta }_{i}^{p}\right)+\sum_{i=1}^{k}\left({\overline{x}}_{i}^{np}-{\overline{x}}_{i}^{p}\right)({\beta }_{i}^{np}-{\beta }_{i}^{p})$$

Here, the first, second and third components on the right side of the equation provided endowments effect, coefficients effect and interaction effect, respectively.

Detailed decomposition was performed to determine the relative contribution of each independent variable to each of the three component (endowments, coefficients and interaction). This involved substituting variables levels/coefficients of one group with those of another group in a sequential manner while keeping the rest of the variables in the equation constant [31, 41]. All analyses were performed in Stata 16.0 (StataCorp; College Station, Texas, USA) using *oaxaca* command for linear regression models [31]. Since the decomposition estimates for categorical explanatory variables rely on the choice of the omitted base category, the command allowed us to apply deviation contrast transform to dummy variables sets so that the contribution of categorical predictors to the ‘unexplained component’ could be identified [31].

## Results

### Descriptive summary

Table 1 presents the background characteristics of the study sample of under-five children included in this study. Mean HAZ for the non-poor and poor groups was -1.15 and -1.59, respectively. The difference in mean HAZ between these groups was statistically significant (*p* < 0.001). There was no statistical difference in the mean age of children (about 30 months) between the non-poor and poor groups. Similarly, there was no statistically significant difference in the distribution of sex of the children between the non-poor and poor groups. The difference in the distribution of children between non-poor and poor was statistically significant for the remaining variables: age of mother at birth, education of mother, number of under-five children in household, type of toilet facility, ethnic group, residence and province.

**Table 1:** Descriptive summary of the study sample of under-five children, Nepal MICS 2019 (*N* = 6277)

Variables	Mean (Std. err) or frequency (%)		*P*-value
	**Non-poor**	**Poor**	
**Height-for-age z-scores (HAZ)**	-1.146 (0.04)	-1.593 (0.04)	< 0.001
**Child’s age (months)**	30.38 (0.35)	30.35 (0.32)	0.784
**Child’s sex**			
Female	1755 (46.6)	1216 (48.4)	0.279
Male	2011 (53.4)	1295 (51.6)	
**Mother’s age at birth**			
< 20 years	611 (16.2)	522 (20.8)	< 0.001
20–34 years	2972 (78.9)	1820 (72.5)	
> 34 years	183 (4.9)	169 (6.7)	
**Maternal education**			
No formal education	695 (18.5)	866 (34.5)	< 0.001
Primary education (grade1-5)	474 (12.6)	488 (19.4)	
Secondary education (grade 6–10)	1496 (39.7)	927 (36.9)	
Higher secondary and above (grade 11 and above)	1101 (29.2)	230 (9.2)	
**Number of under-five children in household**			
One	2464 (65.4)	1360 (54.1)	< 0.001
Two	999 (26.6)	933 (37.2)	
Three	208 (5.5)	206 (8.2)	
Four or more	95 (2.5)	12 (0.5)	
**Type of toilet facility**			
Improved	3593 (95.4)	2215 (88.2)	< 0.001
Unimproved	173 (4.6)	296 (11.8)	
**Ethnic group**			
Brahmin, Chhetri and Madhesi	1730 (45.9)	955 (38.0)	< 0.001
Janajati and Newar	1207 (32.1)	892 (35.5)	
Dalit and Muslim	423 (11.2)	552 (22.0)	
Others (eg. Marwadi, Bangali)	406 (10.8)	112 (4.5)	
**Residence**			
Rural	896 (23.8)	1314 (52.3)	< 0.001
Urban	2870 (76.2)	1197 (47.7)	
**Province**			
Province 1	553 (14.7)	436 (17.3)	< 0.001
Madhesh	1067 (28.3)	381 (15.2)	
Bagmati	947 (25.2)	240 (9.6)	
Gandaki	317 (8.4)	141 (5.6)	
Lumbini	638 (16.9)	529 (21.1)	
Karnali	36 (1.0)	376 (15.0)	
Sudurpaschim	208 (5.5)	408 (16.2)	
**Total**	**3766**	**2511**	

*Abbreviation*: *Std. err* Standard error, *MICS* Multiple Indicator Cluster Survey

**Fig. 1:**
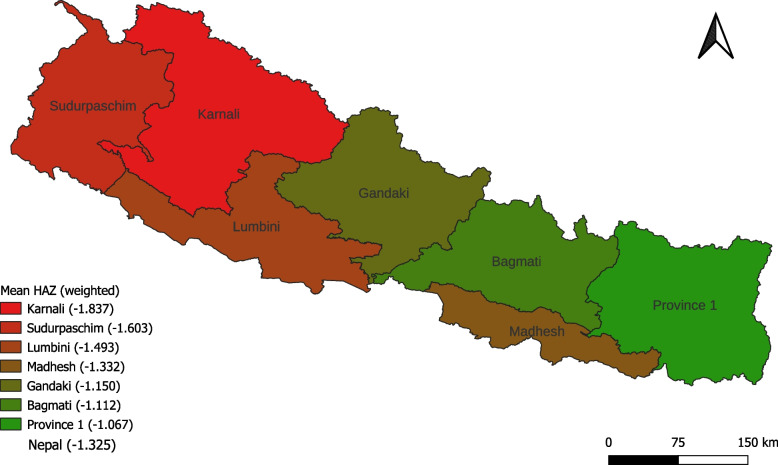
Map of Nepal showing province-wise mean height-for-age z scores (HAZ). Map was created using QGIS 3.22. Shapefile was accessed from publicly available source

Most of the children in both groups had mothers aged 20–34 years. More mothers in the non-poor group had secondary and higher secondary education compared to their poor counterparts. Households in the non-poor group had fewer children compared to the poor group. The improved toilet was more common in households belonging to the non-poor group. More households from the non-poor group belonged to the advantaged caste (Brahmin, Chhetri and Madhesi). Disproportionally more non-poor households were from urban residences. The least number of households from the non-poor group belonged to Karnali province, followed by Sudurpaschim. In contrast, the least number of households from the poor group belonged to Gandaki province followed by Bagmati. The map of Nepal showing the province-wise mean HAZ is given in Fig. 1.

### Results from Blinder-Oaxaca decomposition

Table 2 presents the contribution of different components towards the mean HAZ gap between non-poor and poor groups, obtained from Blinder-Oaxaca decomposition. The mean HAZ gap between the groups was 0.446 (95% confidence interval (CI): 0.341 to 0.554; *p* < 0.001). The group differences in the magnitudes of predictors or covariates (due to endowments) contributed 47% of the mean HAZ gap. Similarly, the group differences in the effects of these predictors (due to coefficients) contributed 68.8% of the mean HAZ gap. Contribution of both components was statistically significant (*p* < 0.001). The contribution of the interaction effect was negative and statistically not significant. The endowments part denotes the average increase in HAZ of children from poor group if they had the same level of predictors as non-poor group. The coefficients part denotes the change in HAZ of children from poor group if they got the coefficients from non-poor group with the current level of predictors. The interaction component provides the simultaneous effect of the disparities in the magnitude of the predictors and coefficients.

**Table 2:** Blinder-Oaxaca decomposition showing mean HAZ difference between non-poor and poor groups and contribution of different components, Nepal MICS 2019 (N = 6277)

	**Estimate**	**Percent contribution**	**95% CI**	***P*-value**
Mean HAZ for Non-poor (group 1)	-1.146		-1.216 to -1.076	< 0.001
Mean HAZ for Poor (group 2)	-1.593		-1.675 to -1.512	< 0.001
Difference	0.447		0.341 to 0.554	< 0.001
Due to endowments (explained component)	0.210	47.0	0.100 to 0.321	< 0.001
Due to coefficients (unexplained component)	0.308	68.8	0.185 to 0.430	< 0.001
Due to interaction	-0.071	-15.8	-0.222 to 0.080	0.359

*MICS* Multiple Indicator Cluster Survey, *CI* Confidence Interval

### Results from detailed decomposition

Table 3 presents the contribution of each variable (covariate/predictor) due to: (i) difference in its distribution between non-poor and poor group towards the value of endowments effect (0.210), (ii) difference in its effect between non-poor and poor group towards the value of coefficient effect (0.308) and (iii) interaction effect (-0.071). Group differences in the level of maternal education between non-poor and poor had the highest contribution (40.7%) towards the gap owing to endowments effect, followed by province (34.3%), residence (16.9%), type of toilet facility (6.1%) and ethnic group (5.8%). Group differences in the magnitudes of these variables disfavored the poor by showing a better endowments effect among the non-poor group. Altogether, these variables explained about 98% of the gap due to endowments effect. Variables with negative signs (such as: child’s age, child’s sex, mother’s age at birth) contributed to narrowing the gap between non-poor and poor groups. Group differences in the effects of ‘number of under-five children in household’ between non-poor and poor had the highest contribution (46.7%) towards gap owing to coefficients effect, followed by ethnic group (14.6%), type of toilet facility (12.2%), maternal age (9.8%), and maternal education (9.6%). Group differences in the effect of these five variables contributed about 93% of the gap due to coefficients effect. For a given number of under-five children in household, a child from non-poor mother benefited from an increased coefficient effect of 46.7% of the total coefficients effect. The largest interaction effect was observed in ethnic group (70.6%), followed by education (27.6%). The negative signs in these variables indicated their contribution to narrowing the nutrition gap between non-poor and poor groups through the interaction effect.

**Table 3:** Blinder-Oaxaca decomposition showing contribution of different variables towards endowments effect, coefficients effect and interaction effect, Nepal MICS 2019 (N = 6277)

Variables	Endowments effect		Coefficients effect		Interaction effect	
	**Coefficient (Std. err)**	**Percent contribution**	**Coefficient** **(Std. err)**	**Percent contribution**	**Coefficient** **(Std. err)**	**Percent contribution**
Child’s age (months)	-0.003 (0.009)	-1.3	0.027 (0.087)	8.8	0.0001 (0.0005)	-0.2
Child’s sex	-0.002 (0.002)	-1.1	-0.001 (0.002)	-0.3	-0.001 (0.002)	1.6
Mother’s age at birth	-0.002 (0.005)	-1.1	0.030 (0.055)	9.8	0.013 (0.008)	-18.7
Maternal education	0.086*** (0.023)	40.7	0.030 (0.022)	9.6	-0.020* (0.031)	27.6
Number of under-five children in household	-0.001 (0.009)	-0.3	0.144* (0.088)	46.7	0.005 (0.014)	-6.5
Type of toilet facility	0.013 (0.01)	6.1	0.037 (0.073)	12.2	0.007 (0.014)	-10.0
Ethnic group	0.012 (0.026)	5.8	0.045 (0.085)	14.6	-0.050* (0.030)	70.6
Residence	0.035* (0.022)	16.9	0.001 (0.003)	0.4	-0.017 (0.029)	23.5
Province	0.072** (0.032)	34.3	0.007 (0.016)	2.4	-0.009 (0.043)	12.1
_cons			-0.013 (0.197)	-4.2		
**Total**	**0.210**	**100**	**0.308**	**100**	**-0.071**	**100**

*Std. err* Standard error, *MICS* Multiple Indicator Cluster Survey

^***^p < 0.001, **p < 0.05, **p ≤ 0.1

Table 4 provides the detailed results from Blinder-Oaxaca decomposition to show the contribution of all the categories (where applicable) of variables towards endowments effect, coefficients effect and interaction effect. The absolute contribution and the percent contribution (for example 0.086 and 40.7%, respectively in case of maternal education) of each variable are further partitioned into the different categories of the variable. The detailed analysis was helpful to identify the categories of variables that contributed most towards the different components of the gap. Regarding endowments effect, the largest contribution was from higher education (28.8%), followed by Karnali (21.6), Sudurpaschim (8.7%), urban/rural (8.2%), no formal education (8.0%), Madhesh (8.0%) and Dalit and Muslim (4.5%). Similarly, regarding coefficients effect, the largest contribution was from single child family (35.0%), followed by maternal age 20–34 years (21.3%), Dalit and Muslim (16.8%), improved toilet facility (14.0%), and two children family (10.3%). Likewise, the largest contribution to the gap due to interaction effect was from Karnali province (36.4%), followed by Dalit and Muslim (35.9%) and higher education of mothers (24.7%).

**Table 4:** Blinder-Oaxaca decomposition showing contribution of all categories of variables towards endowments effect, coefficients effect and interaction effect, Nepal MICS 2019 (*N* = 6277)

		**Endowments effect**			**Coefficients effect**			**Interaction effect**		
**Variable**	**Variable categories**	**Coefficient**	**Std. err**	**Percent contribution**	**Coefficient**	**Std. err**	**Percent contribution**	**Coefficient**	**Std. err**	**Percent contribution**
**Child’s age (months)**	Child’s age (months)	-0.003	0.010	-1.3	0.027	0.087	8.8	0.0001	0.001	-0.2
**Child’s sex**	Female	-0.001	0.001	-0.5	0.015	0.022	4.9	-0.001	0.001	0.8
	Male	-0.001	0.001	-0.5	-0.016	0.023	-5.2	-0.001	0.001	0.8
**Mother’s age at birth**	< 20 years	-0.003	0.003	-1.4	-0.044**	0.021	-14.2	0.010*	0.005	-13.6
	20–34 years	0.000	0.003	-0.2	0.066	0.056	21.3	0.006	0.005	-8.3
	> 34 years	0.001	0.002	0.5	0.008	0.009	2.6	-0.002	0.003	3.1
**Maternal education**	No formal	0.017*	0.010	8.0	0.015	0.032	4.8	-0.007	0.015	9.8
	Primary	0.009*	0.005	4.0	-0.007	0.019	-2.3	0.003	0.007	-3.6
	Secondary	-0.002	0.002	-1.0	0.030	0.029	9.7	0.002	0.003	-3.2
	Higher secondary and above	0.062***	0.015	28.8	-0.008	0.009	-2.6	-0.017	0.019	24.7
**Number of under-five children in household**	One	-0.004	0.009	-1.8	0.108*	0.059	35.0	0.023*	0.013	-31.9
	Two	0.001	0.008	0.7	0.032	0.043	10.3	-0.009	0.012	12.9
	Three	0.001	0.003	0.3	0.005	0.015	1.6	-0.002	0.005	2.4
	Four or more	0.001	0.003	0.7	-0.002	0.001	-0.5	-0.007	0.005	10.1
**Type of toilet facility**	Improved	0.006	0.005	3.0	0.043	0.084	14.0	0.004	0.007	-5.0
	Unimproved	0.006	0.005	3.0	-0.006	0.011	-1.9	0.004	0.007	-5.0
**Ethnic group**	Brahmin, Chhetri and Madhesi	0.006	0.009	3.0	-0.027	0.048	-8.6	-0.006	0.010	7.9
	Janajati and Newar	-0.001	0.004	-0.6	0.031	0.042	10.0	-0.003	0.005	4.3
	Dalit and Muslim	0.010	0.012	4.5	0.052*	0.030	16.8	-0.025*	0.015	35.9
	Others (eg. Marwadi, Bangali)	0.002	0.019	1.0	-0.011	0.014	-3.7	-0.016	0.020	22.5
**Residence**	Rural	0.018*	0.011	8.2	0.015	0.027	4.9	-0.008	0.015	11.8
	Urban	0.018*	0.011	8.2	-0.014	0.025	-4.5	-0.008	0.015	11.8
**Province**	Province 1	-0.009	0.009	-4.0	-0.010	0.020	-3.1	0.001	0.003	-2.1
	Madhesh	0.017	0.018	8.0	-0.007	0.024	-2.1	-0.006	0.021	8.1
	Bagmati	-0.012	0.017	-5.8	0.017	0.012	5.5	0.028	0.020	-39.2
	Gandaki	0.007*	0.004	3.1	-0.011*	0.007	-3.6	-0.006	0.004	9.1
	Lumbini	0.004	0.004	1.9	-0.011	0.021	-3.6	0.002	0.004	-3.1
	Karnali	0.047***	0.012	21.6	0.027	0.025	8.9	-0.026	0.024	36.4
	Sudurpaschim	0.019**	0.009	8.7	0.003	0.019	1.0	-0.002	0.012	2.9
**_cons**					-0.013	0.197	-4.1			
**Total**		**0.210**		**100.0**	**0.308**		**100.0**	**-0.071**		**100.0**

*Std. err* Standard error, *MICS* Multiple Indicator Cluster Survey

^***^p < 0.001, **p < 0.05, **p ≤ 0.1

## Discussion

This study calculated the mean HAZ gap between under-five children belonging to non-poor and poor groups using the most recent nationally representative household survey (MICS 2019). Blinder-Oaxaca technique was employed to decompose the mean HAZ gap into three components: endowments, coefficients and interaction. The factors that contributed most to each of the components were identified. The mean HAZ of non-poor and poor groups was -1.146 and -1.593, respectively. The mean HAZ gap between the groups was 0.447. The between-group mean differences in the characteristics of study participants (endowments) contributed 0.210 (47%) towards the mean HAZ gap. Similarly, the between-group differences in the effect of the characteristics (coefficients) contributed 0.308 (68.8%) towards the mean HAZ gap. The interaction contributed -0.071 (15.8%) towards minimizing the mean HAZ gap.

The contribution of the endowments to the mean HAZ gap provided the portion of the gap that could be effectively reduced by improving the level of predictors in the poor group to reduce wealth-related health inequality. The factors that contributed most to the endowments effect were: maternal education, province (Madhesh, Karnali and Sudurpaschim), residence, type of toilet facility and ethnic group. Here, the policy intervention designed to improve the level of predictors may not be sufficient to reduce the non-poor and poor HAZ gap owing to the significant group differences also in the effects of the predictors. The factors that contributed most to the coefficients effect were: number of under-five children in household, ethnic group, toilet facility, maternal age and maternal education. So, there could be other factors playing an important role in narrowing the differences in the effects of the predictors, such as the amount and right mix of food available to children, quality of maternal education, the underlying health and healthcare utilization of mothers, and quality of the household sanitation. Knowledge and behaviour of parents in the preparation of food and use of available toilet facilities could also narrow the gap between groups.

Our findings are consistent with those from India, Iran and Ethiopia [11, 14, 17, 44] which demonstrated a significant gap in child nutrition based on wealth groups using similar methods. Between-group differences in the level of maternal education accounted for the largest contribution to the gap due to the endowments effect. A similar finding was obtained from earlier studies from Ethiopia and Iran [14, 44]. The between-group difference in the effect of maternal education was also observed, meaning that the same level of education had different returns for the non-poor and poor groups. This finding indicates the difference in quality of education available to different wealth groups. Province (Karnali, Sudurpaschim and Madhesh) was responsible for the second-largest contribution to the gap due to the endowments effect. There could be many reasons: low overall socioeconomic development in these provinces, lack of access to healthcare services, low levels of awareness, difficult geographical terrain in Karnali and Sudurpaschim, poor transportation facility and food insecurity in Karnali [8, 45–47]. Residence (urban/rural) was responsible for the third-largest contribution to the gap due to the endowments effect. Urban–rural disparity in child nutrition was also reported by Sharaf and Rashad based on nationally representative surveys from Egypt, Jordan and Yemen [29].

The between-group difference in the level of toilet facility was responsible for the fourth-largest contribution to the gap due to the endowments effect. Our finding corroborates those obtained from studies conducted in Ethiopia and India [11, 14]. Between-group difference in the effect of toilet facility was also observed, meaning that the same level of toilet facility had different returns to the non-poor and poor groups. This finding might be related to a difference in the standard of toilet facilities and hygiene behaviours of parents belonging to different income groups. Ethnicity (Dalit and Muslim) was responsible for the fifth-largest contribution to the gap due to the endowments effect. Dalit and Muslim also accounted for about 17% of the gap due to coefficient effects. Inequality in child nutrition between scheduled caste and the remaining population was also reported by a study conducted in India [4], which found that the gap was both due to differences in the distribution of level of predictors (wealth, education, use of health services) and effect of the predictors. In Nepal and India, caste-based discrimination put Dalit and minority groups in a disadvantaged position which is manifested in the differential health outcomes between groups [4, 48]. In addition, between-group differences in the effect of the number of under-five children in household and the age of mother jointly accounted for about 56% of the gap due to coefficient effects. Given the number of children in a household, poor families are likely to struggle more for adequate food and the right mix of nutrients in comparison to non-poor families [49, 50]. Similarly, given the age of mothers, the underlying health and healthcare utilization could be better for those belonging to the non-poor group compared to their poor counterparts [51].

### Policy implication

The findings of this study could have implications to improve the child nutrition policies in Nepal. The nutrition policies designed to reduce the inequality between income groups should not only focus on improving the level of predictors, but also on the effects of the predictors. There could be broader factors that contribute to narrowing the differences in the effect of the predictors between groups, such as quality of food, quality of education, healthcare utilization, and quality of sanitation. So, such insight may be helpful to improve the current nutrition policies by adding one extra dimension in the design where the policymakers aim to reduce differences between the socioeconomic groups not only by implementing the interventions that improve the level of underlying characteristics but also by implementing the interventions that improve the effect of such characteristics. This study has elicited the relative contribution of each predictor towards narrowing the child nutrition gap both in terms of levels and effects. The evidence presented in this paper could be used to narrow down the nutrition gap between the income groups.

### Strength of this study

The most recent nationally representative household survey from Nepal was used that employed standard methods and tools to collect the data. So, the findings could be generalized nationwide and compared to studies from other countries that used similar survey design. This study is the first of its kind from Nepal that used Blinder-Oaxaca approach to decompose the nutrition gap into different components and calculate the relative contributions of covariates to the outcome gap.

### Limitation of this study

This paper has a few limitations. We could not include maternal and child health characteristics (such as antenatal care visits, place of delivery, birth weight, breastfeeding, newborn danger signs, and obstetric complications) in the model since such data were only available for most recent live birth within two years preceding the date of data collection. Potential predictors of child nutrition such as maternal body mass index were not available in the MICS dataset. Similarly, dietary diversity-related information was available for children aged 6–23 months only. Childhood diseases related information was not included in the model since the information was collected only for two weeks before the survey. In the decomposition analyses, we had assumed that there was no between-groups difference due to unobserved characteristics. Drawing a causal interpretation was not possible due to cross-sectional nature of the survey. Notwithstanding, this study has elicited empirical evidence on factors that contribute towards different components of the child nutrition gap between non-poor and poor using standard econometric methods. So, the findings from this study have policy relevance in designing the interventions aiming to reduce the child nutrition gap between wealth groups in Nepal and countries with similar socioeconomic contexts.

## Conclusion

There was a significant non-poor and poor gap in nutrition outcome in under-five children in Nepal. The variables that contributed most towards the gap due to between-group differences in the levels were maternal education, province (Karnali, Sudurpaschim, Madhesh), residence (rural/urban), type of toilet facility and ethnic group (Dalit and Muslim). The variables that contributed most towards the gap due to between-group differences in the effects were number of under-five children in family, ethnic group (Dalit and Muslim), type of toilet facility, maternal age and education. Decomposition of child nutrition gap into components revealed that narrowing down the inequality between wealth groups does not only depend on improving the level of the predictors (endowments effect), but also on reducing differential effects of the predictors (coefficients effect). Interventions that could increase the effect of the predictors in the poor group (such as quality and standard of education and toilet facility, adequate nutrition, maternal health, special focus on Dalit and minorities) are imperative to reduce the non-poor and poor gap in child nutrition in Nepal. A mix of factors identified under ‘endowments’ and ‘coefficients’ warrant a multisectoral approach to improving children's undernutrition with a focus on both the levels and effects of the predictors. Policymakers should address the socioeconomic differentials in health outcomes so that equitable progress could be made as envisioned in SDGs.

## Supplementary Information

**Additional file 1: Supplementary table 1.** List of candidate variables identified from literature review and available in Nepal MICS 2019 dataset. **Supplementary table 2.** Variance inflation factors (VIFs) of variables included in the Blinder-Oaxaca decomposition model.

## Data Availability

Publicly available data were used that are accessible from the MICS website (https://mics.unicef.org/surveys) upon request.

## Abbreviations

AIC: Akaike’s information criteria
CI: Confidence interval
EA: Enumeration area
HAZ: Height-for-age z scores
LMICs: Low-and middle-income countries
MICS: Multiple indicator cluster survey
SDG: Sustainable development goal
UHC: Universal health coverage
VIF: Variance inflation factor
WHO: World Health Organization
