# Supplementary material for: Poor and non-poor gap in under-five child nutrition: a case from Nepal using Blinder-Oaxaca decomposition approach
Source: BMC Health Serv Res. 2022 Oct 12;22:1245. doi: 10.1186/s12913-022-08643-6 (PMC9559871; doi:10.1186/s12913-022-08643-6)
Supplement: Supplementary file 1 — Additional file 1: Supplementary table 1. List of candidate variables identified from literature review and available in Nepal MICS 2019 dataset. Supplementary table 2. Variance inflation factors (VIFs) of variables included in the Blinder-Oaxaca decomposition model. [file 12913_2022_8643_MOESM1_ESM.docx]

**Supplementary information**

**Poor and non-poor gap in under-five child nutrition: a case from Nepal using Blinder-Oaxaca decomposition approach**

**Supplementary table 1** List of candidate variables identified from literature review and available in Nepal MICS 2019 dataset

| SN | Variable | Measurement | References |
| --- | --- | --- | --- |
| 1 | Height-for-age z-score (HAZ) | Continuous variable measured as per WHO standards | (1,2) |
| 2 | Child’s age | Age of children in months | (3–8) |
| 3 | Child’s sex | Categorized as: female; male | (3,5–8) |
| 4 | Birth order | Categorized as: first; second and third; fourth and above | (5–9) |
| 5 | Preceding birth interval | Categorized as: first birth; two or less years; more than two years | (9–12) |
| 6 | Twin birth | Categorized as: single; multiple | (4,12) |
| 7 | Mother’s age at birth | Categorized as: less than 20 years; 20-34 years; more than 34 years | (5,7–9) |
| 8 | Maternal education | Categorized as: no formal education; primary education (grade1-5); secondary education (grade 6-10); higher secondary and above (grade 11 and above) | (3,5–9) |
| 9 | Number of under-five children in household | Categorized as: one; two; three; four or more | (3,7) |
| 10 | Number of household members | Categorized as: two to five members; six to eight members; nine or more members | (3,7,12) |
| 11 | Source of drinking water | Categorized as: improved; unimproved | (3,6–8) |
| 12 | Type of toilet facility | Categorized as: improved; unimproved | (3,6–9) |
| 13 | Ethnic group | Categorized as: Brahmin, Chhetri and Madhesi; Janajati and Newar; Dalit and Muslim; Others (eg. Marwadi, Bangali) | (5–7) |
| 14 | Residence | Categorized as: rural; urban | (3,6–8) |
| 15 | Province | Categorized as: Province 1; Madhesh province; Bagmati province; Gandaki province; Lumbini province; Karnali province; Sudurpaschim province | (3,5,8,9) |
| 16 | Wealth group | Categorized as: non-poor; poor | (3–6,8,9) |

NMICS= Nepal Multiple Indicator Cluster Survey

**References**

1. O’Donnell O, van Doorslaer E, Wagstaff A, Lindelow M. Analyzing Health Equity Using Household Survey Data [Internet]. Analyzing Health Equity Using Household Survey Data. Washington, D.C.: World Bank Group; 2007. Available from: http://documents.worldbank.org/curated/en/633931468139502235/Analyzing-health-equity-using-household-survey-data-a-guide-to-techniques-and-their-implementation

2. Sharaf MF, Mansour EI, Rashad AS. Child nutritional status in Egypt: a comprehensive analysis of socioeconomic determinants using a quantile regression approach. J Biosoc Sci [Internet]. 2019 Jan 10;51(1):1–17. Available from: https://www.cambridge.org/core/product/identifier/S0021932017000633/type/journal_article

3. Mohammed SH, Muhammad F, Pakzad R, Alizadeh S. Socioeconomic inequality in stunting among under-5 children in Ethiopia: A decomposition analysis. BMC Res Notes [Internet]. 2019 Mar 29 [cited 2022 Feb 3];12(1):1–5. Available from: https://bmcresnotes.biomedcentral.com/articles/10.1186/s13104-019-4229-9

4. Sharaf MF, Rashad AS. Regional inequalities in child malnutrition in Egypt, Jordan, and Yemen: a Blinder-Oaxaca decomposition analysis. Health Econ Rev [Internet]. 2015 Dec 1 [cited 2022 Mar 18];6(1):1–11. Available from: https://healtheconomicsreview.biomedcentral.com/articles/10.1186/s13561-016-0097-3

5. Kumar A, Singh A. Decomposing the Gap in Childhood Undernutrition between Poor and Non-Poor in Urban India, 2005-06. PLoS One [Internet]. 2013 May 29 [cited 2022 Feb 3];8(5):e64972. Available from: https://journals.plos.org/plosone/article?id=10.1371/journal.pone.0064972

6. Joe W, Mishra US, Navaneetham K. Inequalities in Childhood Malnutrition in India: Some Evidence on Group Disparities. J Hum Dev Capab [Internet]. 2009 Nov 1 [cited 2022 Feb 3];10(3):417–39. Available from: https://www.tandfonline.com/doi/abs/10.1080/19452820903048886

7. Bhusal UP, Sapkota VP. Socioeconomic and demographic correlates of child nutritional status in Nepal: an investigation of heterogeneous effects using quantile regression. Global Health [Internet]. 2022 Apr 20 [cited 2022 Apr 21];18(1):1–13. Available from: https://globalizationandhealth.biomedcentral.com/articles/10.1186/s12992-022-00834-4

8. Kumar P, Rashmi R, Muhammad T, Srivastava S. Factors contributing to the reduction in childhood stunting in Bangladesh: a pooled data analysis from the Bangladesh demographic and health surveys of 2004 and 2017–18. BMC Public Health. 2021;21(1):1–14.

9. Huda TM, Hayes A, El Arifeen S, Dibley MJ. Social determinants of inequalities in child undernutrition in Bangladesh: A decomposition analysis. Matern Child Nutr. 2018 Jan 1;14(1).

10. Van De Poel E, Speybroeck N. Decomposing malnutrition inequalities between Scheduled Castes and Tribes and the remaining Indian population. Ethn Heal [Internet]. 2009 Jun [cited 2022 Mar 4];14(3):271–87. Available from: https://www.tandfonline.com/doi/abs/10.1080/13557850802609931

11. Fenske N, Kneib T, Hothorn T. Identifying risk factors for severe childhood malnutrition by boosting additive quantile regression. J Am Stat Assoc. 2011;106(494):494–510.

12. Fenske N, Burns J, Hothorn T, Rehfuess EA. Understanding child stunting in India: A comprehensive analysis of socio-economic, nutritional and environmental determinants using additive quantile regression. PLoS One [Internet]. 2013 Nov 4 [cited 2021 Oct 14];8(11):e78692. Available from: https://journals.plos.org/plosone/article?id=10.1371/journal.pone.0078692

**Supplementary table 2** Variance inflation factors (VIFs) of variables included in the Blinder-Oaxaca decomposition model

| SN | Variable | VIFs |
| --- | --- | --- |
| 1 | **Child’s age** | 1.01 |
| 2 | **Child’s sex** |  |
|  | Male | 1.01 |
| 3 | **Mother’s age at birth** |  |
|  | 20-34 years | 1.32 |
|  | >34 years | 1.37 |
| 4 | **Maternal education** |  |
|  | Primary education (grade1-5) | 1.51 |
|  | Secondary education (grade 6-10) | 2.01 |
|  | Higher secondary and above (grade 11 and above) | 1.89 |
| 5 | **Number of under-five children in household** |  |
|  | Two | 1.12 |
|  | Three | 1.1 |
|  | Four or more | 1.03 |
| 6 | **Type of toilet facility** |  |
|  | Unimproved | 1.08 |
| 7 | **Ethnic group** |  |
|  | Janajati and Newar | 1.44 |
|  | Dalit and Muslim | 1.25 |
|  | Others (eg. Marwadi, Bangali) | 1.17 |
| 8 | **Residence** |  |
|  | Urban | 1.06 |
| 9 | **Province** |  |
|  | Madhesh | 2.05 |
|  | Bagmati | 1.92 |
|  | Gandaki | 1.61 |
|  | Lumbini | 1.78 |
|  | Karnali | 1.76 |
|  | Sudurpaschim | 1.8 |
|  | Mean VIF | 1.44 |
